# Predicting singleton antepartum stillbirth by the demographic *Fetal Medicine Foundation* Risk Calculator—A retrospective case-control study

**DOI:** 10.1371/journal.pone.0260964

**Published:** 2022-01-20

**Authors:** Dana A. Muin, Karin Windsperger, Nadia Attia, Herbert Kiss

**Affiliations:** Division of Feto-Maternal Medicine, Department of Obstetrics and Gynaecology, Comprehensive Centre for Pediatrics, Medical University of Vienna, Vienna, Austria; Universita degli Studi dell’Insubria, ITALY

## Abstract

**Objective:**

To assess the risk of singleton intrauterine fetal death (IUFD) in women by the demographic setting of the online *Fetal Medicine Foundation* (*FMF*) Stillbirth Risk Calculator.

**Methods:**

Retrospective single-centre case-control study involving 144 women having suffered IUFD and 247 women after delivery of a live-born singleton. Nonparametric receiver operating characteristics (ROC) analyses were performed to predict the prognostic power of the *FMF* Stillbirth risk score and to generate a cut-off value to discriminate best between the event of IUFD versus live birth.

**Results:**

Women in the IUFD cohort born a significantly higher overall risk with a median *FMF* risk score of 0.45% (IQR 0.23–0.99) compared to controls [0.23% (IQR 0.21–0.29); *p*<0.001]. Demographic factors contributing to an increased risk of IUFD in our cohort were maternal obesity (*p =* 0.002), smoking (*p*<0.001), chronic hypertension (*p =* 0.015), antiphospholipid syndrome (*p =* 0.017), type 2 diabetes (*p*<0.001), and insulin requirement (*p*<0.001). ROC analyses showed an area under the curve (AUC) of 0.72 (95% CI 0.67–0.78; *p*<0.001) for predicting overall IUFD and an AUC of 0.72 (95% CI 0.64–0.80; *p*<0.001), respectively, for predicting IUFD excluding congenital malformations. The *FMF* risk score at a cut-off of 0.34% (OR 6.22; 95% CI 3.91–9.89; *p*<0.001) yielded an 82% specificity and 58% sensitivity in predicting IUFD with a positive and negative predictive value of 0.94% and 99.84%, respectively.

**Conclusion:**

The *FMF* Stillbirth Risk Calculator based upon maternal demographic and obstetric characteristics only may help identify women at low risk of antepartum stillbirth.

## Introduction

Intrauterine fetal death (IUFD) is a devastating event, which underlies a heterogeneous spectrum of causes, both acute and chronic [1]. Acute events, such as placental abruption, cord accidents, maternal or fetal trauma, are usually unpreventable, yet bear a low recurrence risk in future pregnancies, unless there is a background risk factor, such as a uterine malformation or maternal clotting and bleeding disorder precipitating placental abruption [2–4]. Conversely, chronic events are often accompanied by placental dysfunction leading to hypoxia, fetal growth restriction and ultimately IUFD, if the fetus is not delivered at an appropriate time [5, 6]. Placental dysfunction is often associated with maternal risk factors, such as antiphospholipid syndrome (APS), systemic lupus erythematosus (SLE), diabetes, obesity, vascular disorders as in preeclampsia, as well as maternal age and assisted reproduction [7–9]. Given these circumstances, the recurrence risk for fetal death is up to 22-fold [10, 11]. Whilst these risk factors are practically unmodifiable, fetal death may be prevented if women are subjected to therapeutic intervention, intensified antenatal surveillance, and eventually delivery [12, 13]. After all, from a socio-epidemiological standpoint, we ought to conceive risk of stillbirth as a continuum of “risk degrees”, rather than a dichotomy between “two extremes of exposure” [14].

Prediction models for stillbirths are commonly defined as “models, scores or clinical decision tools” which estimate the risk of adverse perinatal outcome upon certain variables [15]. To date, 69 prediction models for stillbirth have been described in the literature. A recent systematic review has identified maternal age, body-mass-index (BMI), and diabetes as the three most commonly used variables in such prediction tools [16]. The best evidence with consistently strong association with stillbirth is documented for maternal obesity, pre-existing hypertension, and nulliparity.

The *Fetal Medicine Foundation (FMF)* Stillbirth Risk Calculator is an online tool, which was designed to assess the risk of stillbirth in reference to either maternal history, or a combination of first and second trimester measurements, and biomarkers [17–26]. The demographic model is based upon maternal characteristics (i.e., weight, ethnicity, smoking), medical history (i.e., diabetes, chronic hypertension, SLE, APS), and obstetric history (i.e., parity, stillbirth and/or preeclampsia in previous pregnancies).

By the current study, we aimed to apply the demographic model of the *FMF* Stillbirth Risk Calculator in our cohort of women having suffered IUFD and matched live births as an independent dataset for external validation of this online prediction tool.

## Methods

### Study design and data collection

We retrospectively reviewed all cases of singleton IUFD and live birth that were delivered at our tertiary referral center between January 2003 and December 2019. Inclusion criteria were singleton IUFDs above 21^+0^ gestational weeks with documented fetal and maternal characteristics either at antenatal booking or at the time of delivery. Exclusion criteria for IUFDs were multiple pregnancies, medical or surgical terminations of pregnancy, perinatal fetal deaths, and cases with missing fetal and maternal demographics and medical history.

IUFD cases, which fulfilled the study’s inclusion and exclusion criteria, were matched with singleton live births for fetal sex, maternal age, gravidity, parity, and gestational age at delivery (S1 Appendix).

Maternal and fetal characteristics were retrieved from the electronic database ViewPoint Version 5.6.28.56 (General Electric Company, Solingen, Germany). Medical and obstetric history, including type 1 and 2 diabetes and respective treatment (i.e., diet, insulin, metformin), chronic hypertension, SLE, APS, and previous preeclampsia, were manually retrieved from the women’s medical records. Data were transferred into a study-file sheet, reviewed for accuracy, and made anonymous. Demographic variables from the included subjects were manually typed into the online *FMF* Stillbirth Risk Calculator (https://fetalmedicine.org/research/assess/stillbirth). For this study, we applied the "*Maternal history*” setting only. The individual risk score directly correlates with the degree of risk in each case and is presented in percent (%) or fractions (i.e., 1 in x).

### Definitions

Maternal age was defined as age in years at the time of the delivery. Maternal weight (in kg) was obtained at the first visit. BMI was grouped as underweight (≤18.5 kg/m^2^), normal weight (18.6–24.9 kg/m^2^), pre-obesity (25.0–29.9 kg/m^2^), obesity class I (30.0–34.9 kg/m^2^), obesity class II (35.0–39.9 kg/m^2^), and obesity class III (≥40.0 kg/m^2^). Ethnicity was self-reported by the pregnant woman at the first visit and categorized for this study into white, black, South Asian, East Asian, and mixed. Smoking was defined as current smoker or non-smoker at antenatal booking or at the time of stillbirth. Gravidity was defined as the number of the current pregnancy. Parity was defined as the number of previous deliveries above 24^+0^ gestational weeks or with a fetal weight ≥500 g.

As per Austrian law, each case of fetal death underwent thorough pathology assessment. Upon receipt of all post-mortem results (i.e., fetal autopsy, genetic testing, placental histology, and maternal investigation), we concluded the cause of fetal death according to the taxonomy as described by Reddy et al [27]. Cause of fetal death was defined as the “initial, demonstrable pathophysiological entity initiating the chain of events that has irreversibly led to death” in recognition of potentially multiple competing risks and events, and was categorized according to the *“Causes of death and associated conditions”* (CODAC) classification [28].

### Statistical analyses

The distribution of data was analyzed using the Kolmogorov-Smirnov test. Normally distributed data are expressed as mean ± standard deviation. Not normally distributed variables are expressed as median and minimum-maximum, or 25% and 75% interquartile range (IQR). Categorical data are given as absolute (n) and relative frequencies (%). Continuous data were compared with unpaired t-test and Wilcoxon signed-rank test, respectively. Categorical data were compared with Chi^2^ and Fisher’s Exact test, respectively, with a 99% Confidence Interval (CI). Univariable and multivariable logistic regression analyses were used to determine the odds ratio (OR) with a 95% CI from maternal characteristics and medical history provided for the prediction of stillbirth. Nonparametric receiver operating characteristics (ROC) analyses with an area under the curve (AUC) were performed to predict the prognostic power of the risk score, and to generate a cut-off value that best discriminated between the event of stillbirth versus live birth at the point where the sum of sensitivity and specificity was the highest [29].

Considering a prevalence of 0.3% for IUFDs in Central Europe [30], the positive predictive value (PPV) and negative predictive value (NPV) of the FMF prediction tool was calculated as follows: PPV = (sensitivity x prevalence)/[(sensitivity x prevalence) + ((1–specificity) x (1-prevalence))], and NPV = (specificity x (1–prevalence))/[(specificity x (1–prevalence)) + ((1–sensitivity) x prevalence)].

All reported *p*-values are two-sided, and the level of significance was set at <0.05. Statistical tests were performed by SPSS Statistics Version 26.0.0.0 (IBM Corporation, Armonk, NY, USA), and figures were designed by GraphPad Prism 9 for macOS Version 10.14.6 (GraphPad Software, LLC).

### Details of ethical approval

The study was approved by the Ethics Committee of the Medical University of Vienna (registration number 1759/2018) and complied with the principles outlined in the Helsinki Declaration of 1975, as revised in 2013. Women’s written consent was not required as per the Austrian Federal Act (Protection of Personal Data Regulation, §46, Paragraph 1; 2000).

## Results

### Baseline characteristics

Our study cohort consisted of 144 cases of fetal death and 247 matched live births. Compared to matched controls, women after IUFD had a greater BMI (*p =* 0.002), were more often smokers (*p*<0.001), and suffered more frequently medical conditions, such as hypertension (*p =* 0.015), APS (*p =* 0.017), and diabetes (*p*<0.001), with a more frequent need for insulin (*p* = 0.006; Table 1).

**Table 1:** Maternal and fetal characteristics as presented by the matching variables and variables required to calculate the risk of stillbirth by the *Fetal Medicine Foundation* stillbirth Risk Calculator.

Characteristics				Live births (n = 247)	Stillbirths (n = 144)	*p*-Value
**Matching variables**	Maternal age *(years)*^a^			30.4±6.8	30.9±6.8	0.287 ^c^
	Gravida *(n)* ^b^			2 (1–10)	2 (1–12)	0.210 ^d^
	Para *(n)* ^b^			1 (1–8)	1 (0–9)	0.148 ^d^
	Gestational age at delivery *(days)* ^b^			223 (148–290)	221 (148–290)	0.517 ^d^
	Fetal sex	Male		138 (55.9%)	73 (50.7%)	0.322 ^e^
		Female		109 (44.1%)	71 (49.3%)	
***FMF* Stillbirth Risk Calculator**	**Maternal characteristics**	Maternal Weight *(kg)*		60 (33–119)	65 (42–123)	0.047 ^d^
		BMI *(kg/m*^*2*^*)* ^b^		22.37 (15.7–40.8)	23.64 (15.6–45.3)	**0.002** ^d^
		Ethnicity	White	186 (75.3%)	124 (86.1%)	0.050 ^f^
			Black	11 (4.5%)	7 (4.9%)	
			East Asian	1 (0.4%)	1 (0.7%)	
			South Asian	12 (4.9%)	2 (1.4%)	
			Mixed	37 (15.0%)	10 (6.9%)	
		Smoking during pregnancy		8 (3.2%)	**34 (23.6%)**	**<0.001** ^d^
	**Medical history**	Diabetes	Type 1	12 (4.9%)	1 (0.7%)	**<0.001** ^f^
			Type 2	2 (0.8%)	**22 (15.3%)**	
		Diabetes Treatment	Diet	2 (0.8%)	5 (3.5%)	**0.006** ^f^
			Insulin	12 (4.9%)	**17 (11.8%)**	
			Metformin	0 (0.0%)	0 (0.0%)	
			None	0 (0.0%)	1 (0.7%)	
		Chronic HTN		5 (2.0%)	**10 (6.9%)**	**0.015** ^f^
		SLE		0 (0.0%)	0 (0.0%)	**.**
		APS		1 (0.4%)	**5 (3.5%)**	**0.017** ^f^
	**Obstetric history**	Nulliparous		79 (32.0%)	58 (40.3%)	0.097 ^e^
		Parous		168 (68.0%)	86 (59.7%)	
		Stillbirth in previous pregnancy ^g^		5 (3.0%)	**15 (17.4%)**	**<0.001** ^f^
		Preeclampsia in previous pregnancy ^g^		7 (4.2%)	3 (3.5%)	0.793 ^f^

***Abbreviations*:** *APS*, Antiphospholipid syndrome; *BMI*, Body Mass Index; *FMF*, Fetal Medicine Foundation; *HTN*, hypertension; *SLE*, Systemic lupus erythematosus.

^a^ Mean ± Standard deviation.

^b^ Median (minimum-maximum).

^c^ Unpaired t-test with a level of significance <0.05.

^d^ Mann-Whitney U test with a level of significance <0.05.

^e^ Chi^2^ test with a level of significance <0.05.

^f^ Fisher’s Exact test with a level of significance <0.05.

^g^ In multiparous women only.

The IUFD cohort involved 73 (50.7%) male and 71 (49.3%) female fetuses between 21^+1^ and 41^+3^ gestational weeks at a median age of 31^+4^ gestational weeks. The median fetal weight at stillbirth was 1397 g (180–4450 g). Abnormal fetal post-mortem findings and/or prenatally confirmed congenital malformations were reported in 72 (50.0%) cases. Causes of death were confined as placental pathologies in 26 (18.1%) cases; unknown despite thorough investigation or due to lack of important information in 25 (17.4%) cases; fetal conditions in 9 (6.3%) cases; umbilical cord complications in 6 (4.2%) cases; maternal conditions in 4 (2.8%) cases, and IUFD due to infection in 2 (1.4%) cases.

The matched live birth cohort consisted of 138 (55.9%) male and 109 (44.1%) female newborns delivered between 21^+1^ and 41^+3^ gestational weeks at a median age of 31^+6^ gestational weeks. Median weight at delivery was 1880 g (341–4540 g) with a median Apgar score of 8 points at 1 minute, and 9 points at 5 and 10 minutes, respectively, with a median arterial pH of 7.29 (6.89–7.46). From the five matched live births under 22^+0^ gestational weeks, C*ase 1* was noted to have a univentricular heart (male, birth weight 482g, Apgar 1/1/1). *Case 2* had a preterm delivery following premature rupture of membranes (female, 354g, Apgar 2/1/1). *Case 3* had bilateral kidney agenesis with oligohydramnios (male, 341g, Apgar 1/1/1. *Case 4* suffered amniotic infection followed by preterm delivery (male, 440g, Apgar 1/0/0), and *Case 5* had commissural agenesis and vermis hypoplasia (male, 541g, Apgar 2/1/1). None of those extremely premature newborns survived the first two weeks of life.

### Prediction of stillbirth

The results of univariable and multivariable regression analyses to predict the risk of antepartum stillbirth in our cohort are shown in Table 2. In univariable regression analysis, the risk of stillbirth was higher in women with type 2 diabetes, nulliparous women, and those with a history of stillbirth. In multivariable regression analysis, the risk of stillbirth increased with chronic hypertension, nicotine consumption, and diabetes.

**Table 2:** Univariable and multivariable logistic regression analyses for the prediction of antepartum stillbirth by maternal characteristics and medical history in the pregnant study population (n = 391) at the Medical University of Vienna, Austria.

	*Univariable*		*Multivariable*	
*Variable*	*OR (95% CI)*	*p-Value*	*OR (95% CI)*	*p-Value*
Ethnicity				
Caucasian (*Reference*)	1		1.18 (0.97–1.42)	0.091
Black	0.96 (0.36–2.53)	0.925		
South Asian	0.25 (0.06–1.14)	0.073		
East Asian	1.50 (0.09–24.21)	0.775		
Mixed	0.41 (0.19–0.85)	0.016		
Cigarette smoker	0.14 (0.07–0.29)	<0.001	7.50 (3.22–17.47)	<0.001
Chronic hypertension	0.29 (0.10–0.84)	0.014	4.52 (1.29–15.78)	0.018
APS/SLE	0.12 (0.01–0.99)	0.017	6.84 (0.68–68.53)	0.102
Diabetes mellitus				
None (*Reference*)	1.00		3.13 (1.90–5.15)	<0.001
Type 1	0.16 (0.02–1.25)	0.081		
Type 2	21.18 (4.90–91.58)	<0.0001		
Parity				
Multiparous (*Reference*)	1.00		8.06 (0.19–333.96)	0.272
Nulliparous	0.80 (0.61–1.04)	0.098		
Stillbirth				
No history of stillbirth (*Reference*)	1.00		0.15 (0.05–0.48)	<0.001
History of previous stillbirth	6.89 (2.41–19.67)	<0.0001		
Preeclampsia				
No history of preeclampsia (*Reference*)	1.00		1.85 (0.38–9.05)	0.448
History of preeclampsia	0.83 (0.21–3.30)	0.793		

***Abbreviations*:** *APS*, Antiphospholipid syndrome; *SLE*, Systemic lupus erythematosus.

### *Fetal Medicine Foundation* stillbirth risk score

**Fig 1:**
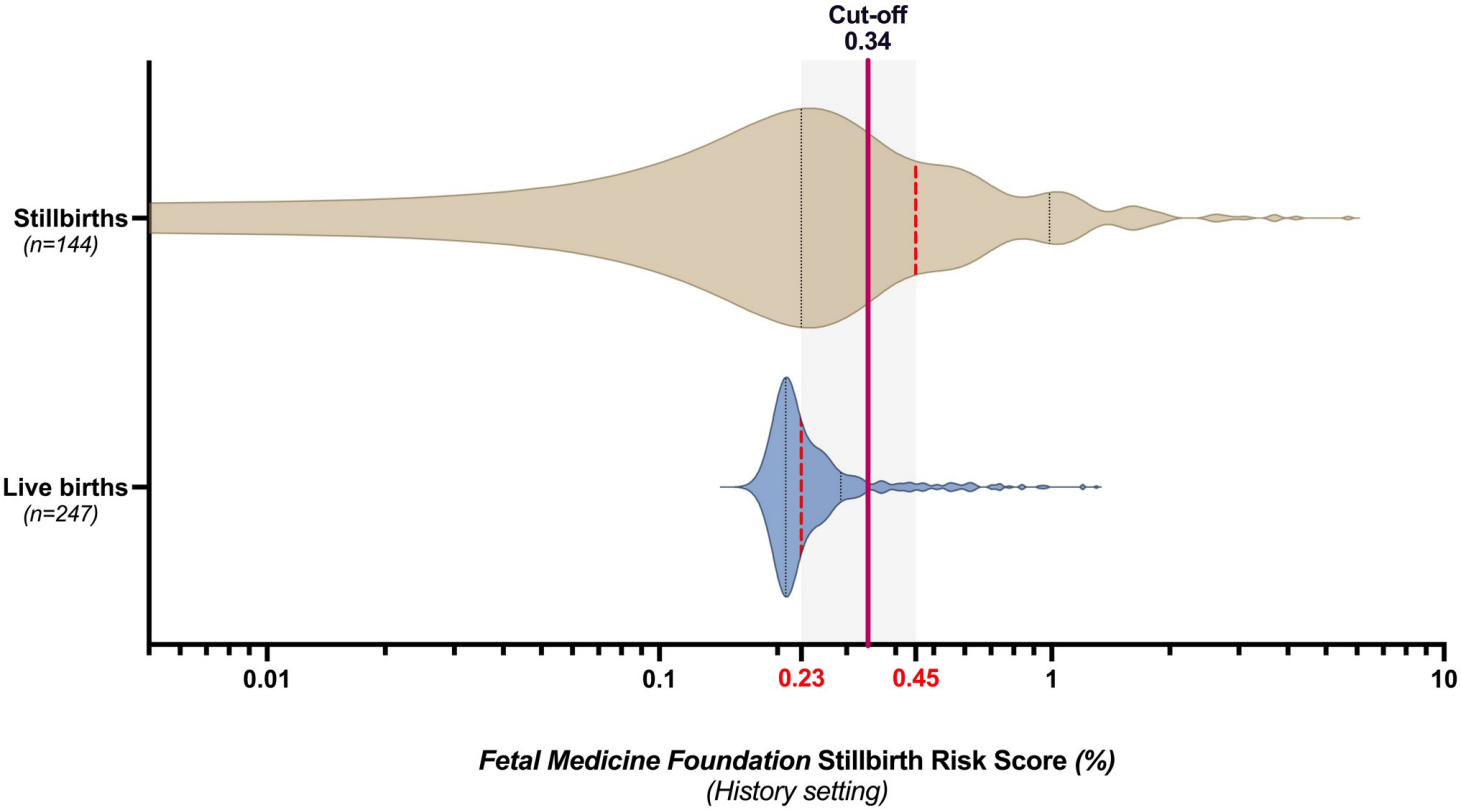
Distribution of the *Fetal Medicine Foundation* stillbirth risk score (in %) in fetal deaths (n = 144) and controls (n = 247). Red line at 0.34% represents the cut-off value to predict IUFD with a sensitivity of 58% and a specificity of 82%. Red dotted lines represent median values of the *FMF* risk score; black dotted lines represent 25% and 75% interquartile ranges; x-axis in Log 10 scale.

Fig 1 shows the distribution of the *FMF* risk score in the IUFD cohort and matched controls. The median risk score in the IUFD cohort was 0.45% (i.e., risk 1 in 222; IQR 0.23–0.99), whilst the median score in the group of matched live births was 0.23% (i.e., risk 1 in 435; IQR 0.21–0.29%; *p*<0.001].

**Fig 2:**
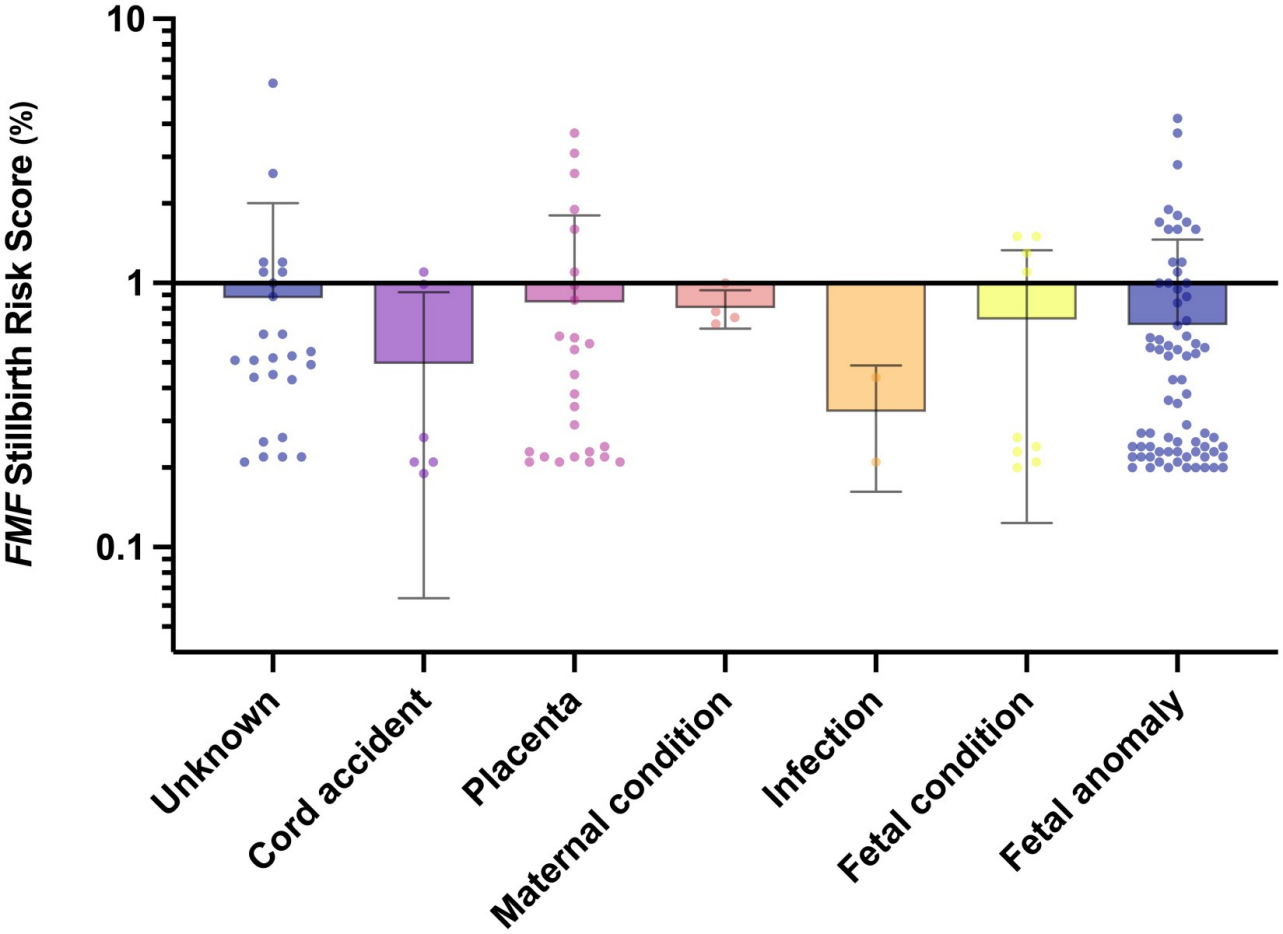
Distribution of the *Fetal Medicine Foundation (FMF)* stillbirth risk score per causes of fetal death (Scatter dot plot with line at mean and standard deviation; y-axis in Log 10 scale).

Fig 2 shows the *FMF* Stillbirth risk scores stratified per causes of fetal death.

To evaluate the discriminative power of the *FMF* risk score we performed a ROC analysis and calculated an AUC of 0.72 (95% CI 0.67–0.78; *p*<0.001) for predicting antepartum stillbirth in the total cohort. The cut-off level of 0.34% yielded a sensitivity of 57.64% (95% CI 49.47–65.41) and specificity of 81.78% (95% CI 76.49–86.10) in identifying risk of IUFD (OR 6.22; 95% CI 3.91–9.89; *p*<0.001; likelihood ratio 3.16) with a PPV of 0.94% and NPV of 99.84%.

**Fig 3:**
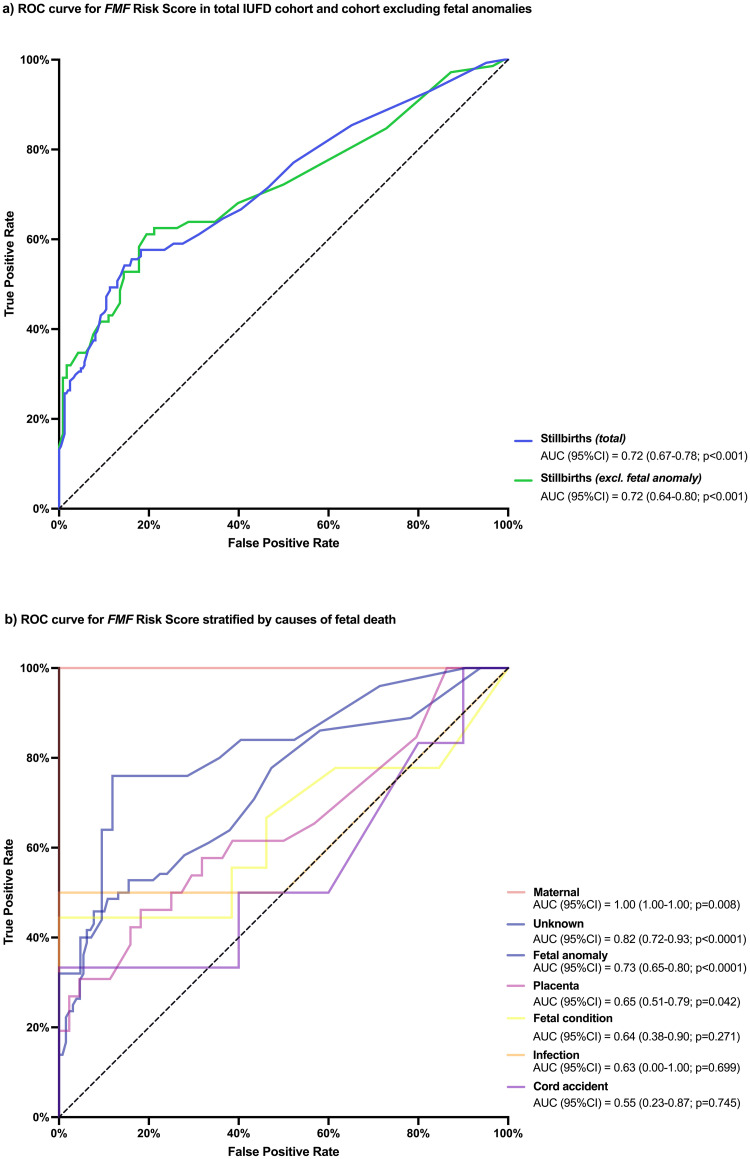
a) Receiver operating characteristics (ROC) curve for the *Fetal Medicine Foundation (FMF)* Stillbirth risk score to predict intrauterine fetal death (IUFD). ROC was performed in the total IUFD cohort (*n = 144*; blue line) and the IUFD cohort excluding cases of fetal anomaly (*n = 75*; green line), respectively. b) ROC curve for the *FMF* risk score to predict IUFD, as stratified by causes of fetal death (maternal *n = 4*; unknown *n = 25*; fetal anomaly *n = 72*; placenta *n = 26*; fetal condition *n = 9*; infection *n = 2*; cord accident *n = 6*).

After exclusion of fetal anomalies (n = 72) from the IUFD cohort, the ROC analysis showed an AUC of 0.72 (95% CI 0.64–0.80; *p*<0.001) with an optimal cut-off at 0.41% to predict IUFD with a sensitivity of 59.72% (95% CI 48.18–70.28) and specificity of 81.36% (95% CI 73.38–87.35; likelihood ratio 3.20) with a PPV of 0.95% and NPV of 99.85% (Fig 3a).

ROC analyses stratified by causes of fetal death showed highest accuracy for prediction of IUFD caused by maternal disease [cut-off 0.72%: sensitivity 75.00% (95% CI 30.06–98.72); specificity 100.00% (95% CI 64.57–100.00)], placental dysfunction [cut-off 0.34%: sensitivity 57.69% (95% CI 38.95–74.46); specificity 68.18% (95% CI 53.44–80.00); likelihood ratio 1.82], fetal anomalies [cut-off 0.34%: sensitivity 52.78% (95% CI 41.40–63.87); specificity 84.50% (95% CI 77.26–89.73); likelihood ratio 3.40], and of unknown etiology [cut-off 0.38%: sensitivity 76.00% (95% CI 56.57–88.50); specificity 88.10% (95% CI 75.00–94.81); likelihood ratio 6.38; Fig 3b].

## Discussion

### Main findings

In this study, we independently assessed and validated the *FMF* Stillbirth Risk Calculator based upon demographic characteristics in a Middle-European case-matched cohort involving IUFDs ≥ 21^+0^ weeks. The online prediction tool achieved similar performance as in the reference group [17], and maternal, medical, and obstetric history yielded a high specificity and satisfactory sensitivity in discriminating between the outcomes live birth versus stillbirth, especially in cases of IUFD due to maternal disease, placental dysfunction, fetal anomaly, and of unknown cause. Most relevant conditions increasing the risk of IUFD in our cohort were type 2 diabetes, previous stillbirth, chronic hypertension, smoking, APS, and SLE.

As the NPV of the demographic *FMF* prediction tool was high (99.8% for the total IUFD cohort and excluding congenital anomalies), the clinical value of the demographic setting lies in identifying women who are at small risk of antepartum stillbirth and providing reassurance that these women do not require close antenatal monitoring, medical interventions or hospital admission. The inclusion of the demographic *FMF* Risk Calculator as early as at antenatal booking may facilitate efficient triage of women and help allocate resources of antenatal surveillance until timely delivery may be required.

Due to the retrospective design of this study, women in our cohort had not been risk-evaluated by the online *FMF* prediction tool in the antenatal or peripartum period. Therefore none of the women had undergone any specific intervention, other than the routine management of their known medical and obstetric conditions according to the best evidence at that time.

### Clinical implications

In the 1980s, the British epidemiologist Geoffrey Rose coined the term *Prevention Paradox* by proving that “a large number of people at a small risk may give rise to more cases of disease than the small number who are at high risk" [31]. Leveling off somewhere between a “high-risk” and “population-based” strategy, there lie the strategies to prevent fetal deaths in high-income countries. With respect to the diversity of the underlying pathomechanisms, the risk of stillbirth is considered a condensation of multiple accumulating risk factors. Reducing the risk of stillbirth therefore requires primarily the collection of maternal, fetal and obstetric characteristics, which may then allow the identification of variables connected with adverse perinatal outcome with highest sensitivity and specificity. Given the relatively low prevalence in Central Europe, yet high cost of false-positive prediction of antepartum stillbirths, it is therefore recommended to choose a prediction cut-off at the lower part of the ROC curve to maximize specificity [32].

It is well recognized, that maternal demographic characteristics may influence fetal outcome. The “history only” setting of the *FMF* Stillbirth Calculator utilizes those demographic patterns and calculates the individual risk based upon a reference population. Whilst it is largely acknowledged that, e.g., maternal diabetes, SLE, APS, sickle cell disease, increased age and previous preeclampsia or stillbirth may hazard the current pregnancy, these women should be naturally considered as high-risk patients by their obstetricians and treated as such with timely intervention when required [13]. Yet, at the same time, the *FMF* Stillbirth Calculator allows objectifying and quantifying this risk in each individual case and may facilitate clinical decision-making regarding close antenatal surveillance and monitoring. With a cut-off risk score of 0.34%, or 1 in 294, women in our cohort were identified as high-risk for placental dysfunction, and stillbirth, likewise. This information may endorse the initiation of e.g. calcium or aspirin during the first trimester or at least enroll the woman into a close surveillance program with regular fetal growth scans, utero-placental Doppler ultrasound measurements, and computerized antenatal cardiotocography [12].

Recent systematic reviews reveal the superiority of maternal characteristics in risk-evaluation [13, 16]. They confirm a robust body of evidence for the link of nulliparity, pre-existing hypertension, and increased maternal BMI to antepartum stillbirth [16, 33]. The demographic findings from our cohort support these data, as all but nulliparity were significantly more prevalent in women affected by fetal death.

In acknowledgment of the challenge to predict adverse events in the presence of competing risks at later stages in pregnancy, the demographic maternal patterns, which may remain unaltered most often during pregnancy, may assist clinical risk assessment at the first booking appointment. Whilst these demographic high-risk characteristics are well recognized among clinicians, it may be useful to provide women with precise risk-estimates, which this readily available and free online tool provides on the basis of over 113 400 singleton pregnancies.

Two characteristics in our cohort are of note: First, we included both stillborn and live-born fetuses with congenital anomalies. Despite this variation to the reference cohort, the prediction model remained accurate with and without the consideration of congenital anomalies in our population. A recent secondary analysis of a case-control study confirmed that among stillbirths, 23.4% had one or more major anomalies compared to 4.3% of live births [34]. Yet, taking these data together, it seems justified to prospectively use this model for risk stratification in stillbirth at preconception, thus ahead of any eventual detection of fetal congenital anomalies later in pregnancy. Also, we considered all IUFD cases from gestational week 21^+0^ onwards. One reason for this is the international heterogeneity in definitions of stillbirth by gestational age and to address those who define fetal death as of week 20^+0^ [15].

### Research implications

Townsend et al. have proposed that a future robust risk prediction tool for stillbirth should incorporate the following candidate variables: maternal age, BMI, parity, pre-existing hypertension, diabetes, previous stillbirth, nicotine consumption, uterine artery Doppler, pregnancy-associated plasma protein PAPP-A, and placental growth factor PlGF [16]. The merit of such a clinical model would be twofold: primarily, the accurate discrimination of high- from low-risk pregnant women, and secondarily, recognizing the variables that may require early enough alteration, if they are modifiable. Whilst maternal age, parity, previous stillbirths, and biomarkers cannot be adapted by intervention, maternal weight, hypertension, type 2 diabetes, and nicotine consumption can be improved through lifestyle modifications [12]. To extrapolate this concept to the demographic model of the *FMF* Stillbirth Risk Calculator, only four variables may be potential subjects to change and therefore possible risk reduction (weight, smoking, diabetes, hypertension). As with many other risk-adjustment models, however, social and behavioral variables, such as domestic abuse, stress, employment, and deprivation are hard to capture and should be further considered within a population-based conceptual framework [35]. Additional research into prediction models may objectify the true preventability of stillbirth by adaption of modifiable risk factors in the future.

### Strengths and limitations

The strengths of this study are first, the strict inclusion and exclusion criteria in the cohort of singleton antepartum stillbirths, each of which was subject to extensive post-mortem investigations to define the cause of death. Our institutional database on antepartum stillbirths includes valid and accurate data, which are regularly checked and updated. This ascertains a continuous degree of data accuracy. The single-center setting of the study also reduces bias and heterogeneity in reporting pathology findings and collection of pregnancy data.

However, we acknowledge the relatively small sample size due to the study design and its retrospective setting. Also, our cohort is ethnically non-diverse, which may further limit the generalizability of the results. Lastly, certain maternal parameters (e.g., smoking, parity, previous preeclampsia) were self-reported by the woman at the time of antenatal booking or delivery and therefore subject to recall bias and potential language barrier in case of foreign nationality, which we cannot control for.

## Conclusion

Raising awareness for maternal epidemiological risk factors is an important measure in antenatal care. The demographic setting of the online *Fetal Medicine Foundation* Stillbirth Risk Calculator may be a useful tool to identify women at low risk of antepartum stillbirth. Its implementation into clinical practice as early as in the preconception period might support obstetrical counseling and further antenatal management. Prospective validation in larger multinational cohort studies is recommended.

## Supporting information

S1 Appendix Flow diagram on the selection of fetal death cases and matched controls. (DOCX)
