# Supplementary material for: Predicting singleton antepartum stillbirth by the demographic Fetal Medicine Foundation Risk Calculator—A retrospective case-control study
Source: PLoS One. 2022 Jan 20;17(1):e0260964. doi: 10.1371/journal.pone.0260964 (PMC8775340; doi:10.1371/journal.pone.0260964)
Supplement: S1 Appendix — (DOCX) [file pone.0260964.s001.docx]

**CONTROLS**

**CASES**

*Singleton live births*

*1^st^ January 2013 –*

*31^st^ December 2019*

*n= 13 205*

*Singleton intrauterine fetal deaths*

*1^st^ January 2013 –*

*31^st^ December 2017*

*n= 211*

**MATCHING FACTORS**

(1) Maternal age

(2) Gravidity

(3) Parity

(4) Gestational age at delivery

(5) Fetal sex

n= 211 n= 336

**EXCLUDED**

**due to missing variables:**

n= 44 Maternal weight n= 51

n= 23 Smoking history n= 38

n= 247

n= 144

**Matching frequency**

1:1 50 cases

1:2 87 cases

1:3 5 cases

1:4 2 cases
